# Risk factors of postoperative pancreatic fistula in patients after distal pancreatectomy: a systematic review and meta-analysis

**DOI:** 10.1038/s41598-017-00311-8

**Published:** 2017-03-15

**Authors:** Yun-Peng Peng, Xiao-Le Zhu, Ling-Di Yin, Yi Zhu, Ji-Shu Wei, Jun-Li Wu, Yi Miao

**Affiliations:** 1 Pancreas Institute of Nanjing Medical University, Nanjing, 210029 People’s Republic of China; 2 Pancreas Center, The First Affiliated Hospital of Nanjing Medical University, Nanjing, 210029 People’s Republic of China; 3 Department of General Surgery, The first Affiliated Hospital of Nanjing Medical University, Nanjing, 210029 People’s Republic of China

## Abstract

Postoperative pancreatic fistula (POPF) is a common complication following distal pancreatectomy (DP). However, the risk factors of this complication in patients after DP still remain controversial. The aim of our study is to estimate the association between potential risk factors and POPF. Relevant articles published up to June 21, 2016 were identified via PubMed, EMBASE, Web of Science, and The Cochrane Library. Studies that examined the risk factors of POPF following DP were enrolled. 20 articles (2070 patients) were finally included in this study. The pooled data suggested that patients with soft pancreas, higher Body Mass Index (BMI), blood transfusion, elevated intraoperative blood loss, and longer operative time had a decreased risk for POPF. However, age, gender, malignant pathology, types of stump closure, octreotide therapy, history of diabetes and chronic pancreatitis, splenectomy, multiorgan resection, main duct ligation, preoperative serum albumin levels, PGA felt wrapping, and extended lymphadenectomy could not be regarded as risk factors for POPF. Our analytic data demonstrated that pancreas texture, BMI, blood transfusion, intraoperative blood loss, and operative time were clinical predictor for POPF. This study may assist surgeons to screen patients with high risk of POPF and select appropriate treatment measures.

## Introduction

Distal pancreatectomy is commonly applied for the resection of pancreatic diseases located in the body and/or tail of the pancreas, such as pancreatic ductal adenocarcinoma, cystic neoplasm, neuroendocrine neoplasm, and chronic pancreatitis^1–3^. Although surgical techniques and perioperative managements for DP were significantly improved in past decades, the complications following DP were still great challenges for surgeons specialized in pancreatic surgery, especially postoperative pancreatic fistula^4^.

According to the definition provided by the International Study Group of Pancreatic Fistula (ISGPF)^5^, POPF manifests as a drain (obtained from operatively or postoperatively placed drain) output with amylase content greater than three times the upper limit of the normal level of serum amylase on or after the third postoperative day. Furthermore, ISGPF also divided POPF into three grades, grade A, B, and C^6^. Grade A pancreatic fistula is an asymptomatic fistula; while grade B and C pancreatic fistula are symptomatic fistula which needs therapeutic intervention (such as antibiotics and/or percutaneous drainage for grade B; resuscitation and/or exploratory laparotomy for grade C). Once postoperative patients suffer from grade B or C pancreatic fistula, a series of other severe complications might develop, including intra-abdominal infection, bleeding, and even shock. Therefore, it is urgent to minutely understand the knowledge of POPF, especially the risk factors which were responsible for the occurrence such complication.

As reported in some studies, a large number of factors could heighten the risk of POPF, such as older age, increased BMI, diabetes history, soft pancreas preoperative blood transfusion, elevated intraoperative blood loss, and longer operative time^7–32^. However, the results of these studies were controversial, and the sample size of them was relatively limited. Therefore, we performed this systematic review to further assess the predictive roles of these risk factors for POPF in patients with DP.

## Results

### Study selection

**Figure 1:**
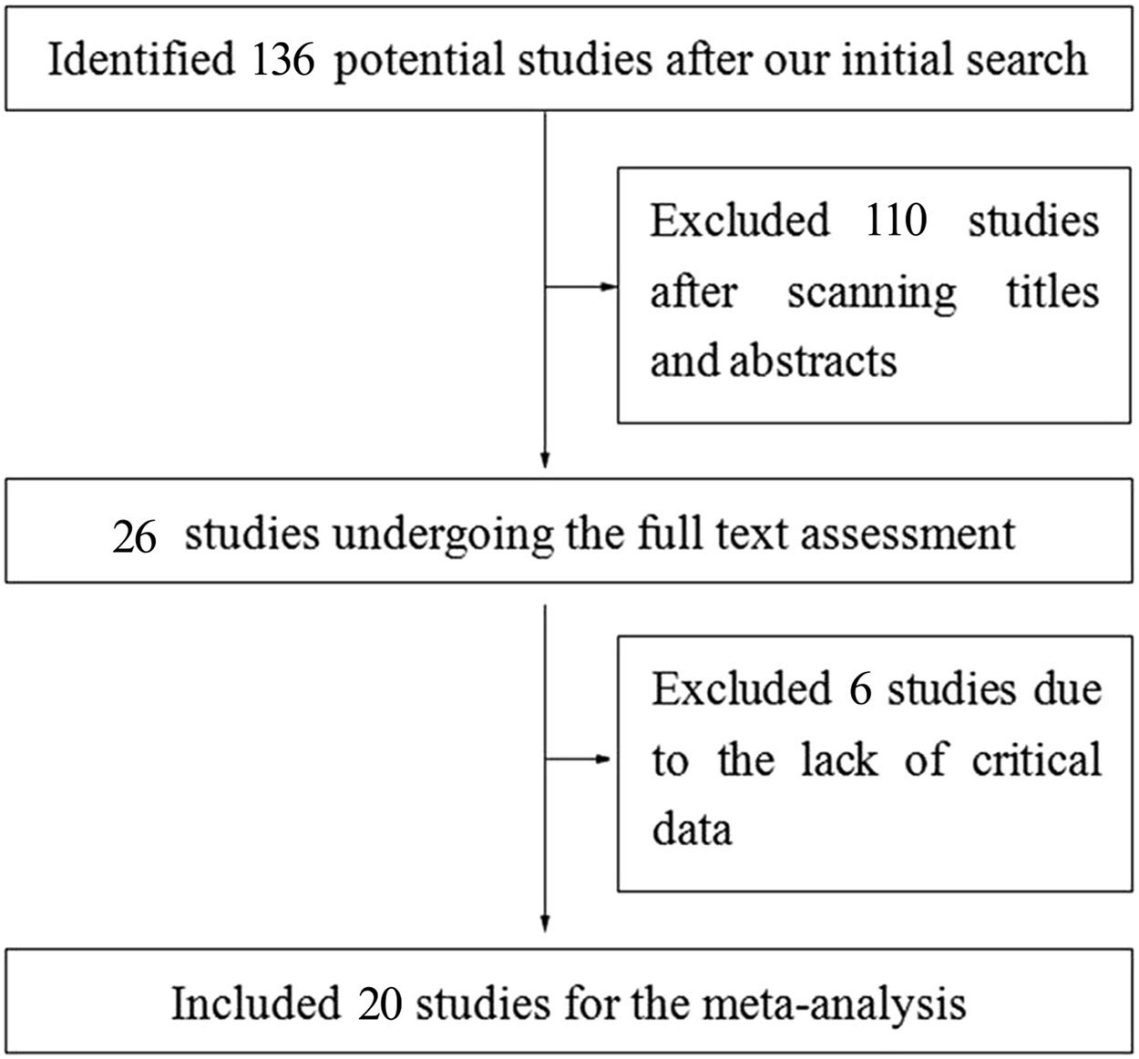
Flow diagram illustrating selection of the articles included in the meta-analysis.

The identification and selection processes for this meta-analysis are illustrated in Fig. 1. A total of 136 potential studies were yielded after our initial search. And then, 110 studies were excluded by scanning titles and abstracts, 26 articles were left for the further full text assessment. According to the inclusion criteria mentioned in materials and methods, 20 articles were finally included to use for this meta analysis. Other articles were excluded due to the reasons as follows: (a) non-focusing on the association between risk factors and POPF after DP; (b) lacking full text; (c) non-providing exact numbers of each group.

### Characteristics of enrolled articles

The main information of these included articles was shown in Table 1. A total of 20 studies with 2070 patients (range from 33–352 per study) were evaluated in this meta analysis^7–25, 32^. All studies were published from 2006 to 2015. Among them, 12 studies investigated the patients from Asian countries, including Singapore (2), Korea (1), India (1), and Japan (8); 8 studies investigated the patients from European countries, North American countries, and North African countries, including Italy (1), France (1), Germany (2), Poland (1), Sweden (1), USA (1), and Tunis (1). 16 of these articles were retrospective studies, 3 of them were prospective studies, and 1 was randomised, controlled trial (RCT). Furthermore, the results of study quality assessment were also listed in Table 1.

**Table 1:** Characteristics of selected studies.

Author	Country	Year	Study design	Definition of POPF	Sample size	Quality assessment
Marco Pericoli Ridolfini *et al*.	Italy	2007	Retrospective	ISGPF	64	7
Brian K. P. Goh *et al*.	Singapore	2008	Retrospective	ISGPF	230	9
Virginie Pannegeon *et al*.	France	2006	Retrospective	NA	175	7
Ryuji Yoshioka *et al*.	Japan	2010	Retrospective	ISGPF	100	7
Marius Distler *et al*.	Germany	2014	Retrospective	ISGPF	124	8
Rachel M Gomes *et al*.	India	2012	Retrospective	ISGPF	33	6
Koji Soga *et al*.	Japan	2011	Retrospective	ISGPF	63	7
Y. Kawabata *et al*.	Japan	2013	Retrospective	ISGPF	40	7
Hidetoshi Eguchi *et al*.	Japan	2011	Retrospective	ISGPF	48	7
Motokazu Sugimoto *et al*.	Japan	2013	Retrospective	ISGPF	106	8
Chiow Adrian Kah Heng *et al*.	Singapore	2009	Retrospective	ISGPF	75	7
Amin Makni *et al*.	Tunis	2012	Retrospective	ISGPF	35	6
Marek Sierzega *et al*.	Poland	2007	Prospective	ISGPF	132	7
Manabu Kawai *et al*.	Japan	2008	Prospective	NA	75	6
Norihiro Sato *et al*.	Japan	2014	Retrospective	ISGPF	44	6
Preeti D. Subhedar *et al*.	USA	2011	Prospective	ISGPF	149	8
Arturo S. Mendoza III *et al*.	Korea	2015	Retrospective	ISGPF	143	8
Keiichi Okano *et al*.	Japan	2011	Retrospective	ISGPF	31	6
Markus K Diener *et al*.	Germany	2011	RCT	ISGPF	352	9
Farshad Frozanpor *et al*.	Sweden	2010	Retrospective	ISGPF	51	7

### The association between risk factors and POPF after DP

#### Non-operation related risk factors

8 non-operation related risk factors were analyzed in this study, including age, gender, BMI, malignant pathology, octreotide therapy, history of diabetes, history of chronic pancreatitis, and preoperative serum albumin levels. All pooled data about these factors were shown in Table 2.

**Table 2:** Pooled data about non-operation related risk factors.

	Number of articles	OR	95% CI	*P*	I^2^(%)	*P*_H_
Age	10	1.35	0.72–2.52	0.350	53	0.030
Gender	16	1.08	0.87–1.35	0.480	0	0.900
BMI	6	2.19	1.35–3.56	0.001	6	0.380
Malignant pathology	15	0.89	0.70–1.12	0.310	28	0.150
Octreotide therapy	5	1.07	0.58–1.99	0.820	51	0.080
History of diabetes	9	0.82	0.59–1.13	0.210	33	0.150
History of chronic pancreatitis	4	1.08	0.45–2.26	0.860	64	0.004
Preoperative serum albumin levels	3	0.51	0.20–1.31	0.160	0	0.980

**Figure 2:**
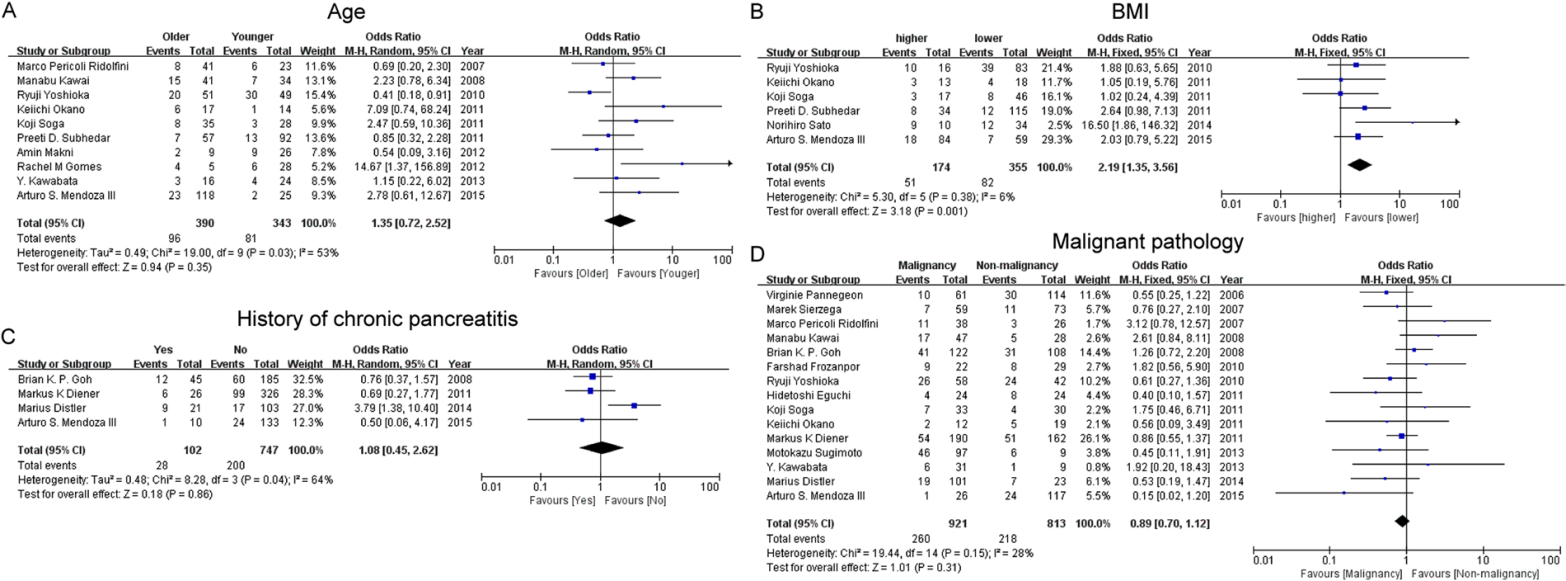
Forest plot of the association between POPF and non-operation related risk factors. (**A**) The association between POPF and age. (**B**) The association between POPF and BMI. (**C**) The association between POPF and history of chronic pancreatitis.

**Age**: 9 of 10 studies suggested that the risk of POPF was not significantly associated with age, while 1 of 10 studies found that increased risk of POPF was observed in older patients. Our pooled data shown that older patients were not prone to suffer from POFP (Table 2, Fig. 2A). However, subgroup analysis suggested that age is a positive factor for POFP according to pooled data from articles published after 2010 and articles with more than or equal to 100 patients (Supplementary Tables 1 and 2).

**BMI:** BMI was mentioned in 6 articles. Although only 1 of 6 articles reported that increased BMI could enhance the incidence of POPF, the combined result supported this finding (Table 2, Fig. 2B), as well as the subgroup results based on the articles with published after 2010, and enrolled more than or equal to 100 patients (Supplementary Tables 1 and 2).

**History of chronic pancreatitis:** 1 of 4 article suggested that patients with chronic pancreatitis had higher risk to suffer from POFP. However, the combined result (Table 2, Fig. 2C) and all subgroup results all showed that there was no significant difference between patients with or without chronic pancreatitis (Supplementary Tables 1 and 2).

**Malignant pathology:** all selected articles and the combined data were reported that patients with malignant tumors do not have an increased risk of POPF (Table 2, Fig. 2D). However, in subgroup analysis, the result showed that malignant tumors were associated with the decreased risk of POPF in studies with larger sample size (≥100) (Supplementary Tables 1 and 2).

**Other factors:** Gender, octreotide therapy, history of diabetes, and preoperative serum albumin levels were respectively reported in 16, 5, 9, 3 enrolled studies. The pooled results illustrated that these non-operation related risk factors mentioned above do not increase or decrease risk of POPF, were in agreement with all articles enrolled in this study (Table 2, Supplementary Fig. 1). Furthermore, the tendency of all subgroup analytic results was in accordance with that of the primary data and combined data (Supplementary Tables 1 and 2).

### Operation related risk factors

10 operation related risk factors were assessed in our study, such as pancreas texture, types of stump closure, blood transfusion, intraoperative blood loss, operative time, splenectomy, multiorgan resection, main duct ligation, PGA felt wrapping, and extended lymphadenectomy. All combined data about factors were shown in Table 3.

**Table 3:** Combined data about operation related risk factors.

	Number of articles	OR	95% CI	*P*	I^2^(%)	*P*_H_
Pancreas texture	14	1.80	1.08–3.02	0.030	51	0.010
Types of stump closure	8	0.75	0.42–1.33	0.330	53	0.040
Blood transfusion	10	1.55	1.11–2.16	0.009	0	0.640
Intraoperative blood loss	8	2.25	1.54–3.29	<0.0001	28	0.210
Operative time	7	1.67	1.08–2.58	0.020	31	0.190
Splenectomy	12	0.91	0.52–1.59	0.730	52	0.020
Multiorgan resection	8	0.77	0.54–1.10	0.150	28	0.210
Main duct ligation	7	0.50	0.24–1.08	0.080	61	0.020
PGA felt wrapping	3	0.75	0.18–3.14	0.690	61	0.080
Extended lymphadenectomy	4	0.82	0.56–1.20	0.300	0	0.820

**Figure 3:**
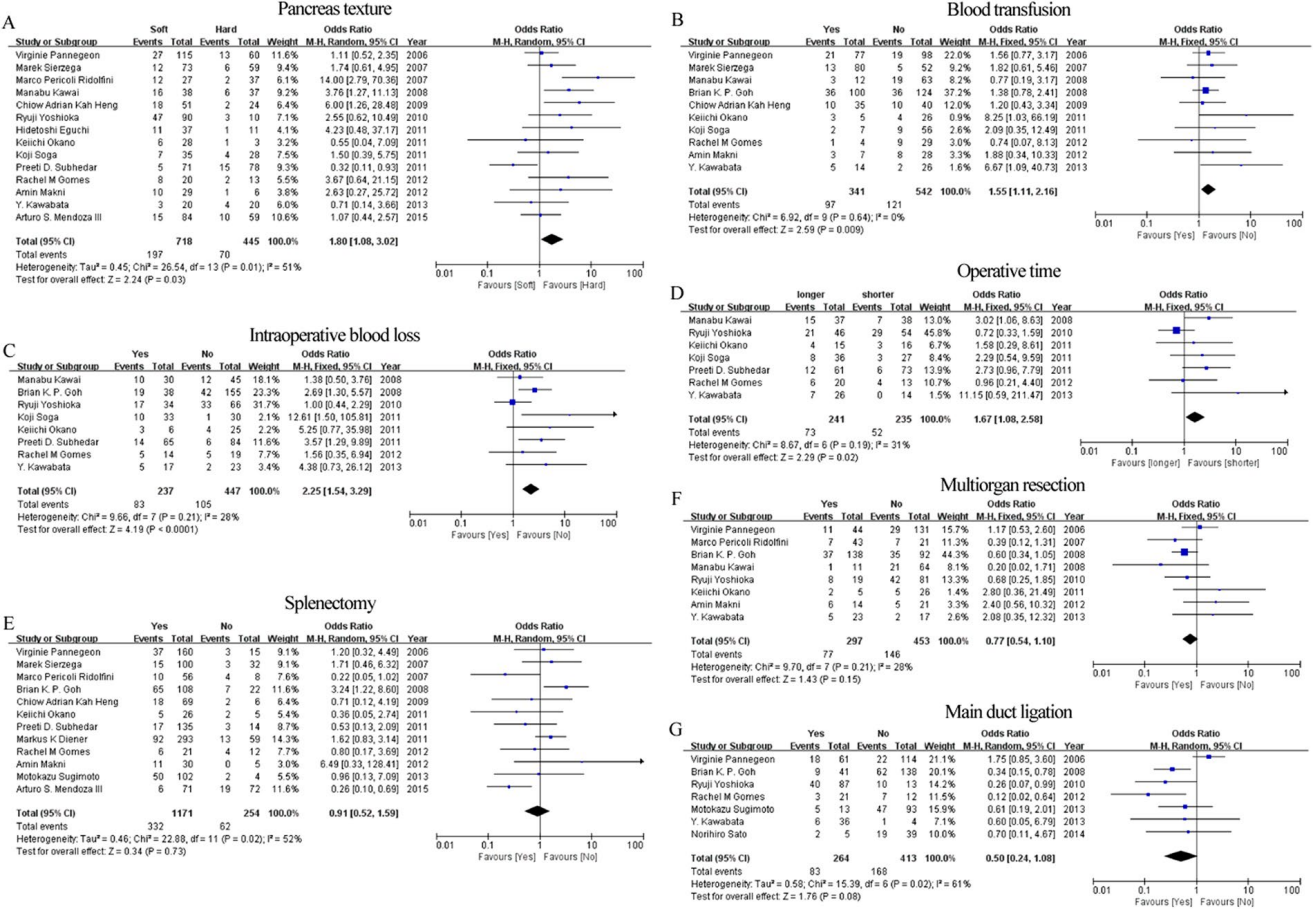
Forest plot of the association between POPF and operation related risk factors. (**A**) The association between POPF and pancreas texture. (**B**) The association between POPF and blood transfusion. (**C**) The association between POPF and intraoperative blood loss. (**D**) The association between POPF and operative time. (**E**) The association between POPF and splenectomy. (**F**) The association between POPF and main duct ligation.

**Pancreas texture**: A total of 14 articles assessed the association between pancreas texture and POPF. 3 of them considered that soft pancreas might increase the risk of POPF, and the pooled OR and 95% CI supported this view (Table 3, Fig. 3A). However, according to the subgroup analysis, we found that similar tendency was just observed in results obtained from studies with larger sample size, and published after 2010 (Supplementary Tables 3 and 4).

**Blood transfusion**: Although only 2 of 10 articles reported that patients with blood transfusion had an increased opportunity to occur POPF, our meta analysis still confirmed this tendency (Table 3, Fig. 3B). Further assessment based on the studies published after 2010 also suggested that blood transfusion could enhance the occurrence of POPF, and other further assessment did not have positive results (Supplementary Tables 3 and 4).

**Intraoperative blood loss**: More intraoperative blood loss might increase the risk of POPF according to the data provided by 3 of 8 articles and the combined data (Table 3, Fig. 3C). All subgroup analysis also showed the coincident results (Supplementary Tabled 3 and 4).

**Operative time**: Our analytic data showed that longer operative time was another promotive risk factors for POPF (Table 3, Fig. 3D). And this result was in accordance with the results provided by Manabu Kawai *et al*., the subgroup results based on articles published after 2010, and articles possessed less than 100 patients (Supplementary Tables 3 and 4).

**Splenectomy:** Brian K. P. Goh *et al*. suggested that DP combined with splenectomy might increase the development of POPF, but Arturo S. Mendoza III revealed the opposite results. However, our assessed results did not agree with them, there was no difference between DP with and without splenectomy (Table 3, Fig. 3E). Moreover, the further analysis based on sample size and the year of articles published all showed that additional splenectomy do not affect the development of POPF (Supplementary Tables 3 and 4).

**Multiorgan resection:** Multiorgan resection was mentioned in 8 articles. Although all articles and pooled data reported that this factor do not influence the incidence of POPF (Table 3, Fig. 3F), the data based on the articles published in or before 2010 suggested that patients with multiorgan resection had a decreased risk of POPF (Supplementary Tables 3 and 4).

**Main duct ligation:** Although 3 of 7 studies agreed that main duct ligation could reduce the incidence rate of POPF, the combined results did not show this tendency (Table 3, Fig. 3G). Moreover, subgroup assessment based on articles published after 2010, and consisted of small sample size also showed this tendency (Supplementary Tables 3 and 4).

**Other factors:** Not only articles enrolled in this study but also our pooled results suggested that other operation related factors did not show any correlation with the development of POPF, including types of stump closure (8 studies), PGA felt wrapping (3 studies), and extended lymphadenectomy (4 studies) (Table 3, Supplementary Fig. 2). In further assessment, subgroup results based on articles published year and sample size suggested that patients with these factors do not had a decreased or increased risk of POPF (Supplementary Tables 2 and 3).

## Discussion

Pancreatic fistula is still a ongoing clinical problem following pancreatic resection, with various technical innovation failing to reduce the occurrence of such complication^33^. Delays in the treatment of POPF will lead to serious problems, such as abscess, pseudoaneurysm, hemorrhage, and sepsis^34–37^. Therefore, in-depth studies focused on the epidemic factors of POPF are urgently needed to provide basis for the prevention and management of POPF. In this study, we reviewed several reported operation and non-operation related risk factors of POPF to further confirm their roles.

To the best of our knowledge, this is the first meta analysis to assess the association between several risk factors and POPF comprehensively. According to the combined ORs and 95% CIs, we confirmed that patients with soft pancreas, higher BMI, blood transfusion, a large number of intraoperative blood loss, and extended operative time were prone to suffer from POPF. Therefore, DP patients with above-named factors should get more attention during the perioperation. Moreover, in subgroup analysis, the predictive role of intraoperative blood loss was consistent in all subgroups, suggesting that effective bleeding control was particularly necessary in DP. However, the positive roles of other risk factors were only observed in some special subgroups. Various reasons might be responsible for this phenomenon. The number of studies in some subgroups were relatively small (such as age, BMI and operative time in articles published before and equal to 2010 group; blood transfusion and operative time in sample size more than 100 group). And well conducted studies with large sample size were lacking (such as BMI in sample size less than and equal to 100 group). Furthermore, we also found that many other risk factors were not associated with the incidence of POPF. The role of some factors might be inconclusive due to the lacking of enrolled studies, such as history of chronic pancreatitis, extended lymphadenectomy, PGA felt wrapping, and serum albumin levels. From the above mentioned, therefore, more studies about some potential articles were greatly needed, especially well designed studies with a large number of patients.

There was significant heterogeneity among the articles included in meta-analysis about age, octreotide therapy, pancreas texture, history of chronic pancreatitis, types of stump closure, splenectomy, main duct ligation, and PGA felt wrapping. To evaluate the reasons of heterogeneity, we performed the subgroup analysis on nationality of patients, year of articles published, and sample size. For age and pancreas texture, the nationality of patients was identified as main factor resulting in heterogeneity; for octreotide therapy, the main factor was sample size; and for types of stump closure and main duct ligation, published year was considered as the main factor. Moreover, the reason of the heterogeneity about history of chronic pancreatitis and PGA felt wrapping was not determined because of the lack of enough articles. To avoid the heterogeneity, further articles were needed which was validated against the main factors. Of course, many other factors also could result in the heterogeneity, such as surgical technology and operation method.

There were also some other limitations in this meta analysis. Firstly, some articles reported the association between risk factors and POPF with ORs and 95% CI were not selected in this study. Secondly, the cut off value of several factors in different articles was not coincidental, such age, BMI, blood loss, and operative time. Thirdly, many other factors might be responsible for the development of POPF. However, they were not assessed in our study due to the lack of sufficient articles or effective data. Finally, the design of eligible articles was highly limited. Almost all of them (16 of 20) were retrospective studies, and more prospective studies and randomized controlled trials were need to improve the reliability of reported data.

In conclusion, this meta-analysis indicates that soft pancreas texture, higher BMI, blood transfusion, massive intraoperative blood loss, and prolonged operative time are markedly associated with the increased incidence of POPF. These findings will provide important theoretical basis for surgeons to overcome the POPF. However, due to the limitations mentioned above, additional well-designed studies with larger sample size are required to confirm the predictive roles of those factors.

## Materials and Methods

### Literature Search

Potential studies were selected by screening PubMed, EMBASE, Web of Science, and The Cochrane Library. The search criteria were ((risk [Title/Abstract]) and distal pancreatectomy [Title/Abstract]) and fistula [Title/Abstract], and ((risk [Title/Abstract]) and left pancreatectomy [Title/Abstract]) and fistula [Title/Abstract]. The last date of retrieval was updated to June 21, 2016. Moreover, the references cited in these articles were also been examined to identify additional relevant studies.

### Inclusion and Exclusion Criteria

Studies that

- included patients underwent distal pancreatectomy or left pancreatectomy;
- investigated postoperative pancreatic fistula;
- assessed the association between risk factors and pancreatic fistula in postoperative patients; and
- were published as a full paper in English were finally enrolled in this meta-analysis.

The following criteria were applied to exclude studies:

- non-human research;
- review articles or letters;
- articles with repeated data; and
- lacking critical data required for further analysis.

### Study Quality Assessment

The quality of the eligible studies was assessed by two independent reviewers according to the Newcastle–Ottawa Quality Assessment Scale for cohort studies (NOS) recommended in the Cochrane Handbook version 5.1.0^38^. The eight elements in the NOS are assessed under three aspects: selection (four elements, one star each), comparability (one element, up to two stars) and outcome (three elements, one star each). The high-quality choices for each element are marked with a star, and then the number of stars is counted to evaluate the quality of each study. Studies are considered high quality if they are awarded six stars or more^39^.

### Data Extraction

All data applied in this study were carefully extracted and estimated by two independent researchers, and any controversial data were re-estimated by both two researchers at the same time. Data extracted from each article were shown as follows:

- article data: author, year of publication, country, study types, numbers of total DP patients, and sample size;
- the definition of POPF and the number of postoperative patients suffered from POPF;
- potential risk factors mentioned in more than or equal to three articles, and the number of patients in each group. Age, gender, BMI, malignant pathology, pancreas texture, types of stump closure, octreotide therapy, history of diabetes, history of chronic pancreatitis, blood transfusion, intraoperative blood loss, operative time, splenectomy, multiorgan resection, main duct ligation, preoperative serum albumin levels, PGA felt wrapping, and extended lymphadenectomy were finally selected, and we will analyze the association between the development of POPF and risk factors mentioned above.

### Statistical Analysis

Review Manager software (version 5.3; Cochrane Collaboration, Oxford, United Kingdom) was applied to perform this meta analysis and provide relative graphics. The numbers of different groups obtained from each article were used to estimate the ORs and 95% CIs, and further to calculate the combined ORs and 95% CIs. Subgroup analysis was performed based on the year of articles published (>2010 *vs*. ≤2010), and the sample size (≥100 *vs*. <100). Cochran’s Q test and the Higgins I-squared statistic were applied to conduct the heterogeneity analysis^40^. Heterogeneity would not be considered significant if the *P*-value for Cochran’s Q test was greater than or equal to 0.1. Random effects model was selected if the *P*-value for Cochran’s Q test was less than 0.1, otherwise fixed effects model was selected^41^. Two-tailed *P*-value was considered statistically significant at less than 0.05.

## Electronic supplementary material

Revised Supplementary data
