# Supplementary material for: Risk factors of postoperative pancreatic fistula in patients after distal pancreatectomy: a systematic review and meta-analysis
Source: Sci Rep. 2017 Mar 15;7:185. doi: 10.1038/s41598-017-00311-8 (PMC5428241; doi:10.1038/s41598-017-00311-8)
Supplement: Supplementary file 1 — Revised Supplementary data [file 41598_2017_311_MOESM1_ESM.pdf]

Risk factors of postoperative pancreatic fistula in patients after distal pancreatectomy: a systematic review and meta-analysis.

Yun-Peng Peng, Xiao-Le Zhu, Ling-Di Yin, Yi Zhu , Ji-Shu Wei, Jun-Li Wu, Yi Miao

Supplementary Data

Supplementary Fig 1

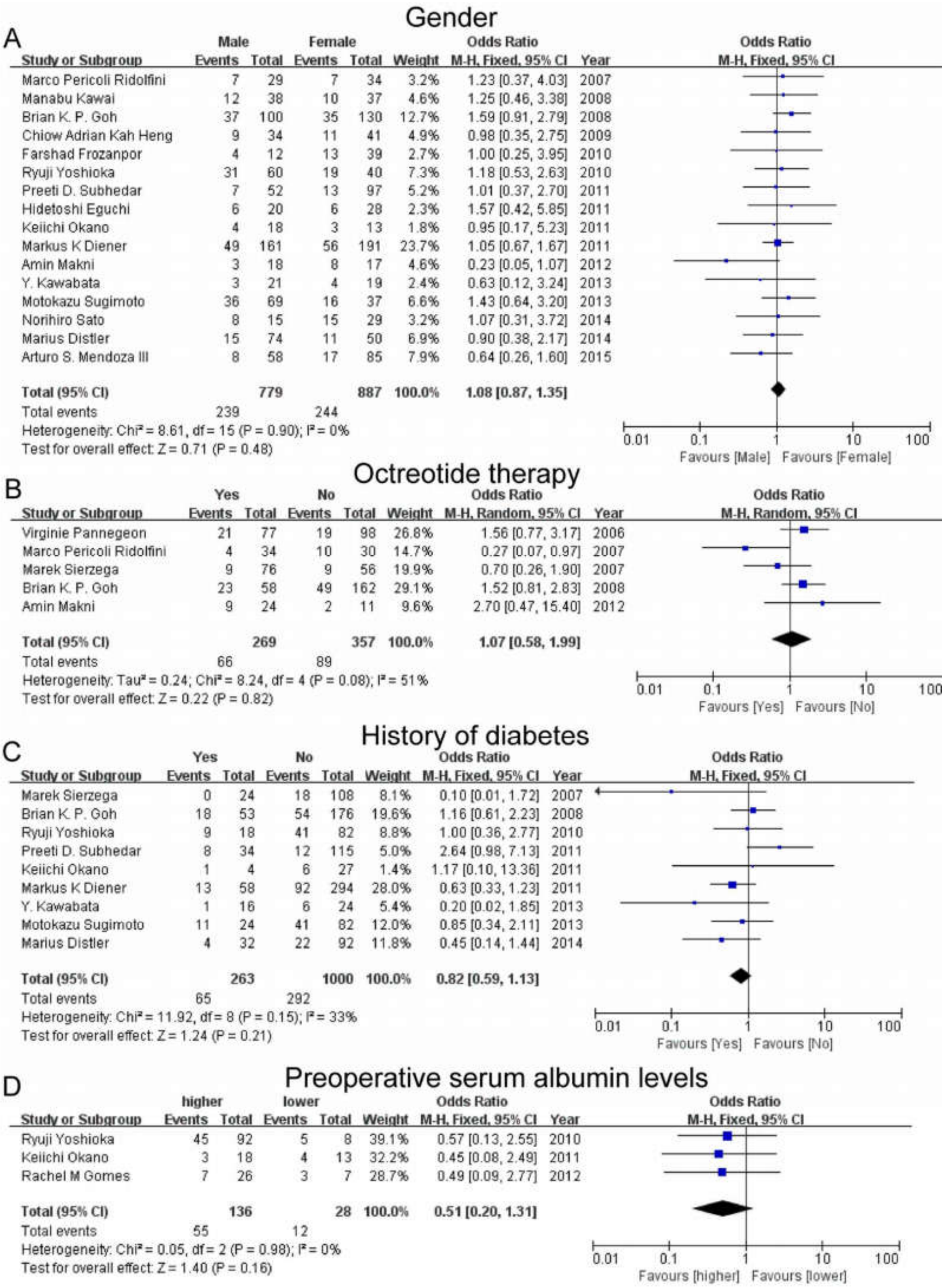

Supplementary Fig 1. Forest plot of the association between POPF and non-operation related risk factors. (A-D) The association between POPF and gender, octreotide therapy, history of diabetes, and preoperative serum albumin levels.

Supplementary Fig 2

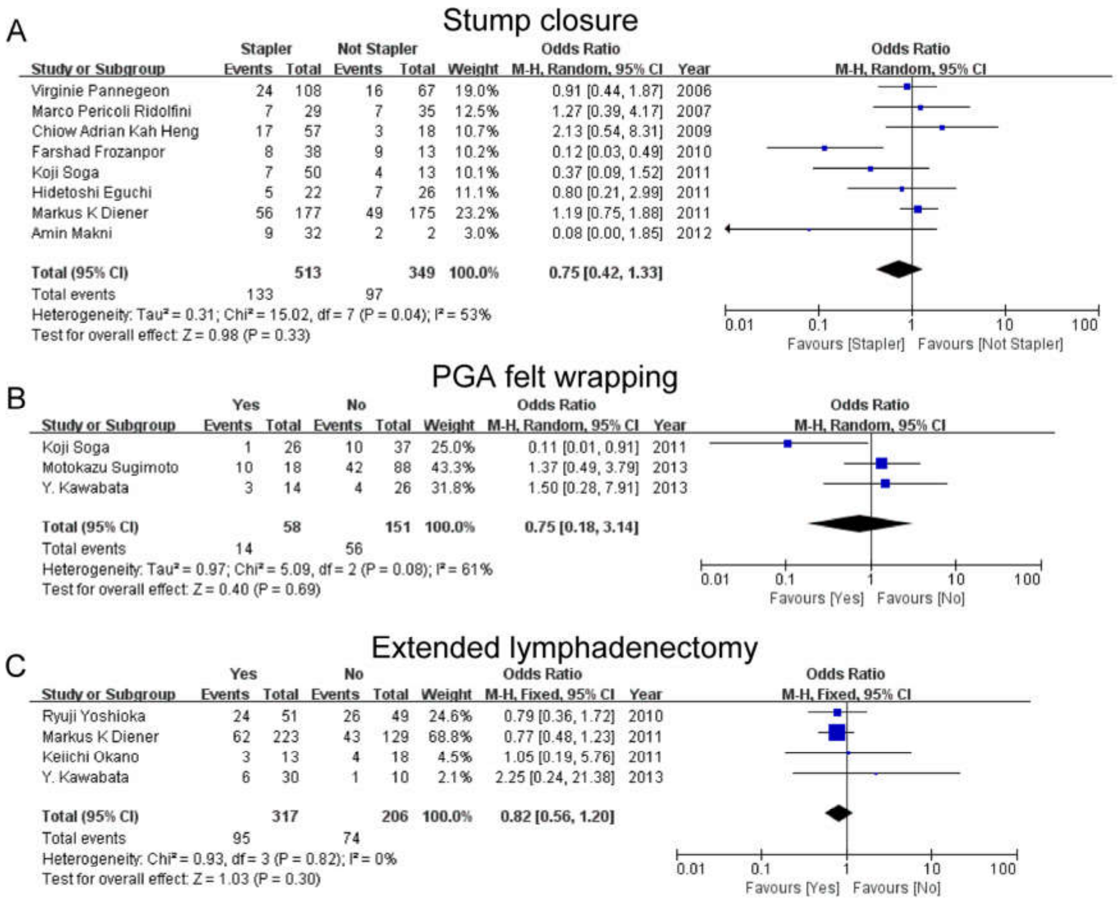

Supplementary Fig 2. Forest plot of the association between POPF and operation related risk factors. (A-C) The association between POPF and stump closure, PGA felt wrapping , and extended lymphadenectomy.

Supplementary Table 1 Subgroup analysis between POPF and non-operation related risk factors according to the year of articles published.

	Number of articles	OR	95% CI	*P*	I^2(%)	P_H
**Age**						
≤2010	3	0.83	0.29-2.35	0.720	69	0.040
>2010	7	1.72	1.01-2.92	0.040	34	0.170
**Gender**						
≤2010	6	1.30	0.91-1.85	0.150	0	0.960
>2010	10	0.96	0.73-1.28	0.800	0	0.740
**BMI**						
≤2010	1	1.88	0.63-5.65			
>2010	5	2.28	1.33-3.91	0.003	24	0.260
**Malignant pathology**						
≤2010	7	1.06	0.78-1.46	0.700	44	0.100
>2010	8	0.72	0.51-1.01	0.060	0	0.460
**Octreotide therapy**						
≤2010	4	0.96	0.49-1.88	0.910	59	0.060
>2010	1	2.70	0.47-15.40			
**History of diabetes**						
≤2010	3	0.89	0.53-1.49	0.660	33	0.230
>2010	6	0.77	0.51-1.17	0.220	42	0.130
**History of chronic pancreatitis**						
≤2010	1	0.76	0.37-1.57	0.460		
>2010	3	1.24	0.33-4.71	0.750	71	0.030
**Preoperative serum albumin levels**						
≤2010	1	0.57	0.13-2.55	0.470		
>2010	2	0.47	0.14-1.58	0.220	0	0.940

Supplementary Table 2 Subgroup analysis between POPF and non-operation related risk factors according to the sample size.

	Number of articles	OR	95% CI	*P*	I^2(%)	P_H
**Age**						
≥100	3	0.84	0.31-2.24	0.730	61	0.080
<100	7	1.76	1.03-2.99	0.040	35	0.160
**Gender**						
≥100	7	1.14	0.88-1.47	0.340	0	0.720
<100	9	0.96	0.63-1.46	0.850	0	0.800
**BMI**						
≥100	3	2.15	1.20-3.86	0.010	0	0.890
<100	3	2.22	0.44-11.32	0.340	61	0.080
**Malignant pathology**						
≥100	8	0.76	0.58-0.99	0.040	8	0.370
<100	7	1.53	0.93-2.53	0.100	13	0.330
**Octreotide therapy**						
≥100	3	1.33	0.87-2.03	0.190	0	0.380
<100	2	0.79	0.08-7.58	0.840	77	0.040
**History of diabetes**						
≥100	7	0.85	0.61-1.18	0.320	40	0.120
<100	2	0.40	0.08-1.95	0.260	10	0.290
**History of chronic pancreatitis**						
≥100	4	1.08	0.45-2.62	0.860	64	0.040
<100	0					
**Preoperative serum albumin levels**						
≥100	1	0.57	0.13-2.55			
<100	2	0.47	0.14-1.58	0.220	0	0.940

Supplementary Table 3 Subgroup analysis between POPF and operation related risk factors according to the year of articles published.

	Number of articles	OR	95% CI	*P*	I^2(%)	P_H
**Pancreas texture**						
≤2010	6	2.87	1.40-5.88	0.004	55	0.050
>2010	8	1.02	0.64-1.63	0.920	28	0.210
**Types of stump closure**						
≤2010	4	0.77	0.28-2.13	0.610	69	0.020
>2010	4	0.98	0.65-1.47	0.910	41	0.170
**Blood transfusion**						
≤2010	5	1.39	0.96-2.00	0.080	0	0.900
>2010	5	2.83	1.26-6.36	0.010	0	0.490
**Intraoperative blood loss**						
≤2010	3	1.63	1.01-2.63	0.010	39	0.200
>2010	5	3.92	2.04-7.55	0.000	0	0.600
**Operative time**						
≤2010	2	1.42	0.35-5.72	0.360	78	0.030
>2010	5	2.29	1.21-4.35	0.010	0	0.620
**Splenectomy**						
≤2010	5	1.13	0.46-2.82	0.790	56	0.060
>2010	7	0.75	0.35-1.59	0.450	51	0.060
**Multiorgan resection**						
≤2010	5	0.65	0.44-0.95	0.030	1	0.400
>2010	3	2.36	0.88-6.38	0.090	0	0.980
**Main duct ligation**						
≤2010	3	0.57	0.16-2.03	0.390	82	0.004
>2010	4	0.42	0.19-0.94	0.030	0	0.400
**PGA felt wrapping**						
≤2010	0					
>2010	3	0.75	0.18-3.14	0.690	61	0.080
**Extended lymphadenectomy**						
≤2010	1	0.79	0.36-1.72	0.550		
>2010	3	0.83	0.53-1.29	0.400	0	0.630

Supplementary Table 4 Subgroup analysis between POPF and operation related risk factors according to the sample size.

	Number of articles	OR	95% CI	*P*	I^2(%)	P_H
**Pancreas texture**						
≥100	5	1.02	0.68-1.55	0.910	45	0.120
<100	9	3.19	1.91-5.34	0.000	23	0.240
**Types of stump closure**						
≥100	2	1.10	0.75-1.62	0.620	0	0.540
<100	6	0.55	0.22-1.43	0.220	59	0.030
**Blood transfusion**						
≥100	3	1.49	0.99-2.25	0.050	0	0.890
<100	7	1.68	0.95-2.96	0.080	9	0.360
**Intraoperative blood loss**						
≥100	3	2.04	1.27-3.28	0.003	56	0.100
<100	5	2.67	1.42-5.03	0.002	20	0.290
**Operative time**						
≥100	2	1.34	0.37-4.93	0.660	75	0.050
<100	5	2.37	1.25-4.51	0.008	0	0.580
**Splenectomy**						
≥100	7	1.08	0.54-2.17	0.820	62	0.010
<100	5	0.68	0.32-1.46	0.320	15	0.320
**Multiorgan resection**						
≥100	3	0.73	0.48-1.12	0.150	0	0.390
<100	5	0.87	0.45-1.70	0.690	47	0.110
**Main duct ligation**						
≥100	4	0.59	0.23-1.53	0.280	74	0.010
<100	3	0.31	0.11-0.90	0.030	11	0.320
**PGA felt wrapping**						
≥100	1	1.37	0.49-3.79			
<100	2	0.44	0.03-6.14	0.540	74	0.050
**Extended lymphadenectomy**						
≥100	2	0.77	0.52-1.16	0.210	0	0.960
<100	2	1.43	0.38-5.35	0.590	0	0.600
